# A rare case of *Listeria monocytogenes* causing mycotic aneurysm of the common femoral artery: A case report

**DOI:** 10.1016/j.ijscr.2019.07.063

**Published:** 2019-07-25

**Authors:** T. Pathmarajah, S. Chu, K. Sieunarine

**Affiliations:** a Department of Vascular and Endovascular Surgery, Royal Perth Hospital, Perth, Australia; b Department of Plastic and Reconstructive Surgery, Fiona Stanley Hospital, Perth, Australia

**Keywords:** Mycotic aneurysm, Listeria, Graft infection, Femoral, Case report

## Abstract

**Introduction:**

Mycotic aneurysms are an uncommon occurrence, with*Staphylococcus* and *Salmonella* species found to be the causative pathogen in up to 95% of cases. We believe this is the first described case of a common femoral artery mycotic aneurysm due to *Listeria monocytogenes.*

**Presentation of case:**

A 66-year-old male presented with a two-month history of an increasing painful mass in his left groin, on the background of immunosuppression treatment for ankylosing spondylitis. He was afebrile on assessment, with a normal white cell count. Contrast enhanced CT scan showed a common femoral artery aneurysm, with no infective features. His aneurysm was excised and repaired with a Dacron tube graft. *L. monocytogenes* was cultured from the aneurysm tissue, and he was commenced on appropriate antibiotic treatment. The prosthetic graft was also replaced with a venous bypass of the aneurysm.

**Discussion:**

*L. monocytogenes* is a rare cause of mycotic aneurysm with less than 40 cases reported in the literature. Immunosuppression is a recognised risk factor for Listerial infections. Immunocompromised patients may not display typical clinical or biochemical features associated with a mycotic aneurysm. Prosthetic graft infections are associated with significant mortality, with excision of the prosthetic material and venous reconstruction associated with good outcomes for eradicating infection.

**Conclusion:**

This report highlights the importance of obtaining intraoperative tissue samples for microbiological and histopathological assessment in immunocompromised patients. This is important for the detection of rare organisms such as *L.monocytogenes*, requiring targeted antibiotic therapy. Inappropriate treatment of Listerial infections can result in serious invasive illness.

## Highlights

• Immunosuppressed patients may not display typical clinical or biochemical features associated with mycotic aneurysms.
• Clinicians should have a high index of suspicion for infective aetiology when treating aneurysmal disease in immunocompromised patients.
• It is important to obtain intraoperative tissue samples for histopathology and microbiological assessment in immunocompromised patients for detection of rare pathogens.
• Autogenous vein should be used in infected surgical fields to avoid the risk of prosthetic graft infection.

## 1 Introduction

Mycotic aneurysms are uncommon events, occurring in 3% of all arterial aneurysms [1]. The most common sites of mycotic aneurysm formation include the abdominal aorta (31%) and the femoral artery (38%) with the latter most commonly caused by iatrogenic or self-inflicted trauma e.g intravenous drug users. Although in the pre-antibiotic era, mycotic aneurysms were originally attributed to infective endocarditis, this is now a rare underlying cause and hematogenous seeding of a previously damaged atherosclerotic vessel is the commonest mechanism of infection. The most common organisms isolated from infected aneurysms are gram positive bacteria, accounting for 60% of cases, most commonly Staphylococci [2]. Gram negative bacteria (chiefly Salmonellae) can be isolated from 35% of cases [2]. Since the first published report of a *Listeria monocytogenes* infected aortic aneurysm in 1965, there have been less than 40 reported cases worldwide [[2], [3], [4], [5]] with majority involving the abdominal aorta. We believe this to be the first described case of a common femoral artery mycotic aneurysm due to *L.monocytogenes*.

This work has been reported in line with the SCARE criteria. [17]

## 2 Case

A 66-year-old male presented to his general practitioner with a two-month history of an increasing painful mass in his left groin associated with medial thigh paresthesia. He denied any fevers, infective symptoms and was otherwise systemically well. His past medical history included ankylosing spondylitis for which he was on long-term prednisolone (5 mg mane), and had commenced Infliximab infusions (400 mg 6-weekly) 10 months prior. Other significant medical history included ischemic heart disease with four coronary stents, and an open repair of an abdominal aortic aneurysm. On assessment he was hemodynamically stable and afebrile, and examination of his left groin revealed a tender pulsatile mass, with no overlying skin changes. Biochemistry revealed a normal white cell count.

**Fig. 1:**
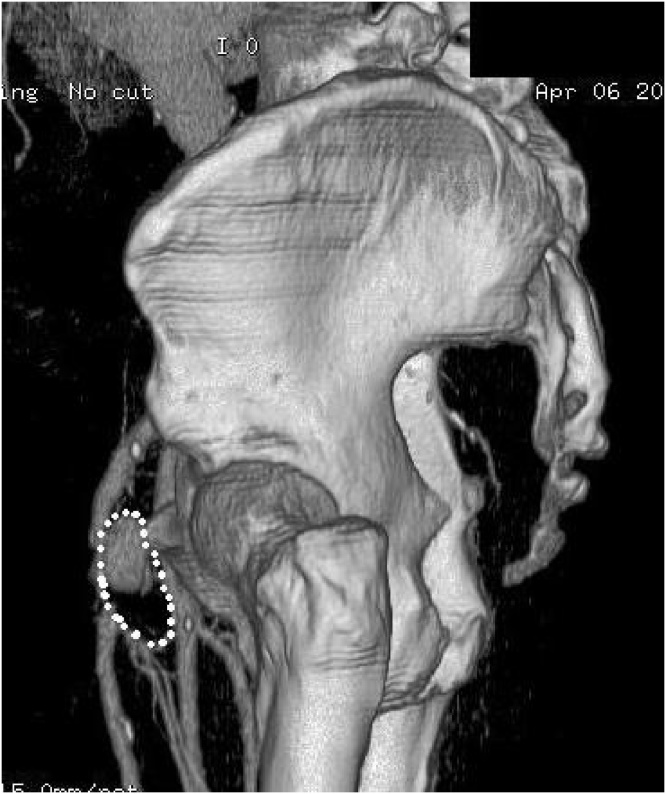
A 3-dimensional reconstruction of the right femoral aneurysm in this patient as shown on CT angiogram. The actual size of the aneurysm including thrombus outlined.

**Fig. 2:**
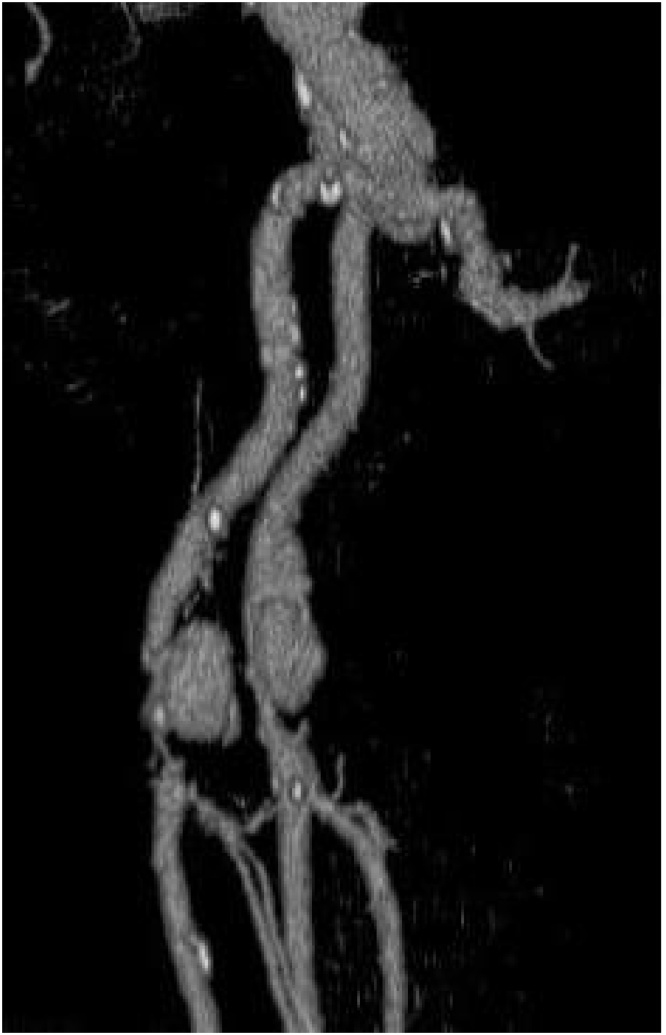
A 3-dimensional reconstruction of the femoral arteries showing the bilateral femoral aneurysms in this patient.

A duplex ultrasound showed a dilatation of the left common femoral artery suggestive of a possible pseudoaneurysm or mycotic aneurysm. This was further evaluated with a CT angiogram of the lower limbs which showed bilateral common femoral artery aneurysms associated with areas of arterial dissection. The left aneurysm extended 5.5 cm cranio-caudally, 4 cm transversely and 3.5 cm antero-posteriorly. (Fig. 1) The left distal common femoral artery was markedly stenosed as it draped over the aneurysm. The right aneurysm was 1.5 mm in maximal diameter and 2 cm in cranio-caudal length with moderate stenosis of the right distal common femoral artery. The CTA did not show any features suspicious for a mycotic aneurysm (Fig. 2)

The patient underwent an uncomplicated resection of the left common femoral artery aneurysm with repair performed using an 8 mm Dacron tube graft. Intraoperatively the vessel wall appeared normal, with no evidence of oedema or suppurative material in the surrounding tissue therefore an infective etiology was not suspected. The aneurysmal tissue specimen was sent for histopathological and microbiological testing. *L. monocytogenes* was cultured from this tissue. Histopathological examination of the aneurysmal wall and contents demonstrated hyaline fibrosis and focal dystrophic calcification accompanied by amorphous debris containing cholesterol crystals, foamy histiocytes, acute and chronic inflammatory cells, focal hemorrhage and neovascularization. There was also inflammation and fibrosis extending into the fatty tissue surrounding the large blood vessel.

Perioperatively the patient was administered 2 g of cephalothin for surgical prophylaxis, with nil further antibiotics continued post operatively. With the isolation of *L. monocytogenes*, the patient was commenced on IV ampicillin 2 g QID and gentamicin 1 mg/kg/tds in accordance with the therapeutic guidelines. Due to the risk of prosthetic graft infection, the Dacron patch was replaced with a venous bypass of the aneurysm in a second operation. Microscopy and culture of the specimens taken at the second operation (femoral wall tissue, groin tissue, left groin swab and graft specimen) showed no residual evidence of *L. monocytogenes*. Post-operatively, the patient was continued on a total of 6 weeks of ampicillin. The contralateral aneurysm was treated at a later stage with excision and venous bypass. Microbiological testing of the aneurysmal tissue again demonstrated *L. monocytogenes,* which was treated in a similar manner with a prolonged course of oral antibiotics post-operatively.

## 3 Discussion

*L. monocytogenes* is a gram positive, non-sporulating, facultatively anaerobic bacillus. The organism is ubiquitous in soil and water and can be isolated in the gastrointestinal flora of animals and up to 5% of healthy human adults. *L. monocytogenes* is able to grow in a wide range of pH as well as at low temperatures; a feature that enables this organism to multiply in food stored at refrigeration temperatures [6,7]. The main route of transmission is via ingestion of contaminated food. Clinical manifestation of *L. monocytogenes* varies from self-limiting febrile gastroenteritis in normal hosts, to invasive disease including meningitis, meningoencephalitis or bacteremia in immunosuppressed adults. Ampicillin or amoxicillin has been the drug of choice in most *L. monocytogenes* infections. Cephalosporins have limited activity against the organism, with some patients progressing to develop listerial meningitis whilst on cephalosporin therapy. [6]

The rates of *L. monocytogenes* infection are highest among infants <1 month of age and adults > 60 years of age. [6] About 70% of all non-perinatal infections occur in immunosuppressed patients with hematological malignancies or AIDs, in transplant recipients and in patients on corticosteroid therapy [6]. Immunosuppressive states are also described as an independent risk factor for mycotic aneurysm formation. In a retrospective review of 43 patients treated for mycotic aneurysms, up to 70% were found to have immunosuppressive disorders [8]. Furthermore, patients with atherosclerosis, particularly older adults, are at risk for bacteremic seeding of atheromatous plaques [9]. In this case the patient had atherosclerotic disease of the arterial wall, and also systemic immunosuppression therefore increasing susceptibility to mycotic aneurysm formation.

Leukocytosis is often seen in up to 70% of patients with mycotic aneurysms, however our patient had a normal white cell count. [10] In a review of 48 patients with infected aneurysms Hsu and Lin described the intraoperative presence of gross pus in 54% of patients, the authors concluding local purulent infection was associated with high risk of prosthetic graft infection and aneurysm related death [11]. Intraoperative examination of the aneurysm in our patient did not raise suspicion for an infective aetiology as there was no gross pus or necrotic tissue around the aneurysm. CT angiography is the most useful imaging modality for diagnosing an infected aneurysm, however no features of perivascular fluid collection, intramural/extravascular gas, or inflammation of the tissue surrounding the vessel was present in our case to suggest infective aetiology [12]. The lack of inflammatory response in our patient is most likely the result of his immunosuppressed state.

The management of infected aneurysms remains a challenge in current practice, with reported hospital mortality rates ranging from 16 to 44%. [13] Almost all untreated aneurysm eventually leads to rupture. No randomized controlled trials exist to guide the treatment of infected aneurysms, with management strategies primarily based upon clinical experience and available case series. The standard accepted treatment of infected aneurysms includes targeted antibiotic therapy in conjunction with surgical debridement with or without revascularization. Chu et al described 45 patients treated for infected mycotic aortic aneurysms, with 35 undergoing surgical debridement of the necrotic tissue and in-situ reconstruction with a prosthetic graft, while 11 were managed with antibiotic therapy alone. One-year mortality rate was 25% in the operative group versus 59% in the medical management group. The authors also noted that 30-day mortality rates were significantly lower in patients undergoing elective surgery in comparison to emergency surgery (0% vs 36%), highlighting urgent surgical intervention in patients with mycotic aneurysm is associated with poor prognosis [14].

A significant concern with in-situ reconstruction with prosthetic graft material is the development of prosthetic graft infection. Prosthetic graft infections occur in 1%–6% of cases after vascular graft implantation procedures [13]. Intraoperative bacterial contamination is cited as the most common cause, with other causes including bacterial colonization of the thrombus or direct inoculation. Prosthetic vascular graft infection is associated with a mortality and amputation rate of up to 70% [13]. Chu et al reported 6 prosthetic graft complications (3 early and 3 late) amongst the 35 patients who underwent surgical debridement, with an associated 100% mortality rate among these patients [14]. Total excision of the infected graft, and extra-anatomical bypass is considered the gold standard of treatment; however is disadvantaged by low patency rate, re-infection of the new graft, and often persistent infection at the site of the vascular stump with blow out and life threatening haemorrhage reported in cases of aortic infection [15]. The use of rifampicin-impregnated grafts and silver coated grafts have been described for anatomical reconstruction, however they are still associated with significant rates of re-infection [16]

The use of autologous vein for arterial reconstruction for graft infection has been well described with good outcomes. Daenens et al described 49 patients with prosthetic graft infection who were successfully treated with autogenous vein reconstruction, with no incidence of reinfection. [16] Ehsan and Gibbons described a 10-year experience of using autogenous deep vein reconstruction for arterial and prosthetic graft infections, including 6 patients with mycotic aneurysm with two involving the femoral artery. The authors concluded autogenous vein was an excellent conduit for arterial reconstruction in the presence of infection, with venous bypass almost eliminating recurrent infection reducing risk of later graft rupture and minimizing mortality [15]. In this case we chose to replace the Dacron patch once infective aetiology was confirmed using an exclusion bypass of the aneurysm to minimize risk of prosthetic graft infection.

## 4 Conclusion

This case highlights the need for a high index of suspicion for an infective aetiology when treating aneurysmal disease in immunocompromised patients. These patients may not display typical clinical or biochemical features seen in non-immunocompromised patients with mycotic aneurysms. It is important to obtain intraoperative specimens for microbiological and histopathological testing for unusual organisms in these patients, as routine antibiotic therapy may not be active against the causative organisms with inadequate treatment potentially resulting in more invasive illness. Furthermore, autogenous vein should be considered in infected surgical fields to avoid the risk of prosthetic graft infection.

## Funding

None.

## Ethical approval

This case report was exempt from ethical approval at our institution.

## Consent

Written informed consent was obtained from the patient for publication of this case report and accompanying images. A copy of the written consent is available for review by the Editor-in-Chief of this journal on request.

## Author contribution

Dr Tishanthan Pathmarajah – Study concept, gathering data, writing the paper.

Dr Sharon Chu – Study concept, gathering data, writing the paper.

Dr Kishore Sieunarine – Study concept, operating surgeon, editing the paper.

## Registration of research studies

Not applicable for this case report.

## Guarantor

Dr Tishanthan Pathmarajah.

## Provenance and peer review

Not commissioned, externally peer-reviewed.

## Declaration of Competing Interest

None.
